# Clinical supervision for clinical psychology students in Uganda: an initial qualitative exploration

**DOI:** 10.1186/s13033-015-0016-8

**Published:** 2015-06-23

**Authors:** Jennifer Hall, Rosco Kasujja, Peter Oakes

**Affiliations:** School of Psychology, Makerere University, Kampala, Uganda; Department of Psychological Health and Wellbeing, University of Hull, Hull, England, UK

**Keywords:** Supervision, Cross-cultural, Uganda, Clinical psychology, Training, Mental health

## Abstract

**Background:**

Burn out in clinical psychologists working in low income countries has been reported. Clinical supervisory structures do not yet exist in Uganda. A way to decrease levels of burn out and increase quality of care for people with mental illness is through clinical supervision. The aim of this study was to explore the initial experiences of supervision for clinical psychology students in Uganda to ascertain whether or not clinical supervision is culturally appropriate, and what aspects of supervision had been helpful and unhelpful.

**Methods:**

A qualitative design with thematic analysis was utilized. A focus group was held with 12 second year clinical psychology students to ask their experiences of receiving supervision.

**Results:**

Data analysis created five themes. Firstly, the negative emotions that resulted from the training processed were discussed, and how supervision helped and did not help the students to manage these. Secondly, the students voiced that supervision helped them to learn through observational experiences, co-therapist roles and parallel processes within the supervisory relationship. Thirdly, supervision had taught the clinical psychology students their role as a clinical psychology student, how to act within the Ugandan mental health system and skills to conduct therapy. Fourthly, suggestions for the future of supervision were given, with the students requesting for it to start earlier in the training, for supervisors who can meet with the students on a regular basis to be selected and for the training the students receive at university to match the skills required on their placements, with a request for more practical techniques rather than theory. The final theme related to left over miscellaneous data, such as the students agreeing with each other.

**Conclusions:**

The students stated that supervision was helpful overall, implying that clinical supervision is culturally appropriate for clinical psychology students in Uganda. Suggestions for future supervision were given. In order to decrease high levels of staff burn out in the mental health systems in Uganda, supervisory structures with an emphasis on self care need to be established.

**Electronic supplementary material:**

The online version of this article (doi:10.1186/s13033-015-0016-8) contains supplementary material, which is available to authorized users.

## Background

Clinical supervision has been defined as “the formal provision by senior/qualified health practitioners of an intensive relationship-based education and training that is case-focussed and which supports, directs and guides the work of colleagues” [1]. Supervision for psychologists was offered in 43.5% of the countries surveyed by the World Health Organisation in 2010 [2]. This survey consisted of 147 countries and included those without clinical psychologists such as Bhutan [2, 3]. The least supervision was offered in Africa where only 28.8% of countries offer supervision for psychologists, and low-income countries were less likely to offer supervision for psychologists than high-income countries [2] perhaps due to the insufficient “human resources to provide such regular supervision” [4]. A country in Africa where human resources in clinical psychology are lacking is Uganda, where a total of four clinical psychologists practice in state-run mental health institutions (J. Nsereko, personal communication, 14th April 2014), catering for the mental health needs of the 34.1 million population [5]. Burn out has been defined as a multidimensional concept, with a core component of emotional exhaustion (the practitioner’s emotional resources have been depleted due to the demands and expectations of the work), and two other components of depersonalization (when responses become excessively negative or detached) and reduced personal accomplishment/professional efficacy (when the practitioner feels inadequate professionally) [6–8]. Clinical supervision has been suggested to significantly decrease levels of burn out among staff working in community mental health services in the UK [9, 10]. Given this, it is not surprising that Ugandan clinical psychologists who are working under exceptional pressures to meet the mental health needs of this country without any clinical supervisory structures in place [11, 12], have reported feeling “burnt out” and “demoralised” [13].

Positive steps to set up clinical psychology supervision in Uganda have been taken by Makerere University, the country’s state-run university, which offers a 2 year Masters of Science Clinical Psychology programme. This training course began in 1998 and traditionally had a large theoretical rather than practical component, with students relying on lecture notes and books to see clients [14]. More recently, practical teachings in cognitive behavioural therapy (CBT) and narrative exposure therapy (NET) have been taught and, in 2012, supervision of clinical placements was introduced. However, minimal research has been conducted into how best to support training and qualified clinical psychologists working in low-income countries through supervision, a gap that this research aims to begin to address.

The existing research into supervision in low-income countries describes training models using supervisors in mental health [15–17] and the role of clinical supervision in decreasing burn out [18]. These papers are of a descriptive nature, with there being a lack of empirical research into how supervision can best support mental health staff, and in particular clinical psychologists working in low income countries such as Uganda. The majority of research into supervision for psychologists has been conducted in high-income countries.

High-income countries where supervision for training clinical psychologists is a pre-requisite for accreditation of clinical psychology training courses include the UK [19–21], America [22] and Australia [23]. A vast amount of research has been conducted into supervision for training clinical psychologists in these countries [24], looking at models of supervision as well as what makes supervision effective. Models of supervision have suggested that effective supervision fulfil three roles: restorative (emotional support), normative (maintaining professional standards) and formative (develop supervisee’s skills) (e.g. [25, 26]) and have looked at the stages of development in supervision (Stoltenberg et al. as cited in [27]**)**. Supervision has been conceptualised in terms of the ‘stages of change’ model [28], has incorporated narrative and positive psychology elements [29] and theories of supervisory relationships has been researched [30].

A wealth of research has looked into supervision from the clinical psychology students’ perspective, suggesting that supervisors should give positive feedback before constructive criticism, acknowledge process and transference, joint problem solving, let supervisees come up with their own ideas, validate the supervisee’s feelings, be reliable (e.g. turn up on time for supervision, answer supervisee’s emails) and offer a “safe space” for supervision [31–36].

The aim of this research was to explore the initial experiences of supervision for clinical psychology students in Uganda. Since no previous research has been conducted into this area, no pre-determined data frameworks were used. This was with the hope of giving a rich description of the students’ experiences.

It was hypothesised that the clinical psychology students would find clinical supervision useful. This was because previous research suggests “Western” therapies and methods of clinical psychology are useful in the Ugandan context (e.g. [37]) and clear gaps had been identified previously by Ugandan clinicians in the restorative and normative aspects of supervision [13]. It was expected that the useful and not so useful aspects of supervision may be different to those described by the Western clinical psychology students. Ugandan health and education sectors are based on the British systems, following the colonial rule from 18951 to 962 [38]. The hierarchy and ethnic division which was imposed on Uganda within this time [39] may still be impacting on Ugandan society, for example it has been stated that a “Doctor is next to God” in Uganda [13]. Due to this, it possible that the Ugandan clinical psychology students may show a preference for a power hierarchy and a need to be “told” what to do. Due to supervision being a new concept, it is possible that the trainees’ answers may not be as in-depth, in particular with regards to the not-so-useful aspects of supervision.

## Method

### Context

A collaboration between Hull University in the UK and Makerere University in Uganda set up a supervisor training for Ugandan-based clinical psychologists in October 2012. This was in response to a need identified by staff at Makerere University. A presentation outlining the importance of supervision in clinical psychology was given at the Ugandan Clinical Psychology Conference, also in October 2012 [40]. Following this, nine Ugandan-based clinical and counselling psychologists voluntarily attended the training course, which started the following week. All supervisors had been trained up to at least a Masters level in either clinical or counselling psychology, with seven of them being Ugandan and the remaining two being European (one English and Spanish).

The training lasted 5 days and followed the curriculum specified for Supervisor training developed by the Group of Trainers in Clinical Psychology in the UK, approved by the British Psychological Society. Second year Masters students were then assigned individual supervisors for the practical element of the course, a placement at the country’s psychiatric inpatient hospital, Butabika. At the end of the first semester, after the students had received 6 weeks of supervision, it was felt that the students had gained enough experience in supervision to share their initial thoughts. A focus group was held in the Department of Psychology at Makerere University in Uganda at the end of December 2012.

### Participants

Second year clinical psychology students who received clinical supervision were asked to partake in the focus group. Out of the 18 second year clinical psychology students, 12 volunteered to be in the focus group (males = 4; females = 8), with the other 6 students having other engagements. Informed consent was sought from all participants partaking in the study, and all were told that their identities would be kept confidential.

A reflective commentary of emerging themes and ideas was kept by one of the authors from the start of the research.

### Focus group questions

Questions and prompts were based on the models of supervision discussed above. The aims of the questions were to explore the student’s experience of clinical supervision and to ascertain whether clinical supervision for students in Uganda is useful and appropriate. Questions asked were as follows:

*How often do you receive supervision?*

*What have been the benefits of supervision?*

*What have been the not so useful aspects of supervision?*

*How has supervision helped you to learn?*

*How has supervision given you emotional support?*

*How has supervision helped you think about your relationship with your clients and your supervisor?*

*Any suggestions for future supervision?*

Open ended group questions and prompts were used to facilitate the discussions. The facilitators were members of staff at Makerere University in Uganda, with whom the students were familiar. The focus group lasted approximately 1 h.

### Qualitative analysis

A qualitative design using focus groups was utilized. The focus group transcript was analysed using a thematic approach, following detailed guidelines and “checklist of criteria for good thematic analysis” [41]. In order to keep an emphasis on the experiences of the students, an inductive “bottom up” approach was taken, whereby all of the data set was included in the analysis without any pre-conceived framework. The first author transcribed the focus group discussion and coded the text into different semantic themes, looking at the dialogue presented rather than the meaning behind the data. An essentialist stance was taken, whereby a simple uni-directional link between language and meanings was assumed. The first author generated initial codes for all of the data set, ensuring equal emphasis was given to all of the data. Additional file 1: Table S1 shows an example of a data excerpt and coding. These codes were then collated and organized into initial themes. These themes and codes were checked against each other and revised until each theme was felt to be unique in its own right, and the coded text to fit within these themes. The overall analysis was then checked and revised again to ensure the themes encapsulated the data set as a whole. This process was not linear, but circular with themes and codes constantly being updated.

## Results

Data analysis created five themes and eleven sub-themes which can be seen in Additional file 2: Table S2.

## Emotions

A large proportion (approx 20%) of the dialogue spoke about emotions, with it being divided into (1.1) the participants’ own emotions surrounding training to be a clinical psychologist and the ways in which supervision (1.2) had and (1.3) had not helped with these emotions.

### Emotions surrounding training to be a clinical psychologist

The participants appeared to find the journey of training to be a clinical psychologist impacted negatively on their emotional wellbeing. They described feeling afraid or low in mood in relation to beginning psychological therapy and starting to receive supervision.

*“I was very tense, actually I told myself I want to do the right thing and you know in front of the supervisor you can make a mistake and you know I was tensed and anxious so she sat in on my session and it went on because I was anxious, very anxious during that session…”*

*“There are times that I would attend supervision when I really downcast and sad and low because of um some sessions or that would be going on but she really helped me build my confidence, I became hopeful…”*

### Ways in which supervision had helped manage these emotions

There was consensus that supervision had generally helped the students manage these negative emotions. The useful methods were grouped into three categories: through offering direct emotional support; promoting self care and through the supervisory relationship.

Helpful direct emotional support was said to be given through discussion with the supervisor. The supervisor offering encouragement, giving the students permission to talk about their emotions and offering practical advice about how to conduct therapy was seen as useful.

*“He [supervisor] would guide me and show me that even at this point I can make it and gave me that strength and more morale. And I’ve been able to put in like my all knowing that I can make it, even at this point”.*

*“Supervision is not all about the patient, it is also about the supervisor being involved in helping us if we are interested…that made us very comfortable.”*

The second method in which supervision helped the students to manage their emotions was through promoting self care.

*“I had a lot of anxiety so she helped me take care of self.”*

Anxiety related to supervision was said to be alleviated through discussion about the supervisory relationship.

*“At first I had that fear of facing him [supervisor] even just the appointment since it was maybe after two weeks I was like ‘eh, if I make it, how will he…’ but in fact the way, in fact he first got feedback from me was ‘how am I handling you?’. Thereafter I found it very easy and I would often go there without, with ease, I don’t fear him anymore…”*

### Impact of not engaging with the emotional content

Supervision had not contained two of the participants’ emotional reactions to their clients. In both of these circumstances, it appeared that the supervisor had correctly responded to the practical elements of their dilemma, however that the emotional element had not been validated or reflected upon. Both students appeared anxious and low in mood when discussing the dilemma.

“*I decided to ask a supervisor, but in this particular time I think probably there was a problem with myself. I, I maybe I wasn’t confident with what I had done or [how] I dealt with the patient…but I still felt like I had to do more and more and more and my supervisor told me that sometimes you have to let the patient go. But I don’t know, I kept having that gut feeling yeah. So I, I’m still there and I’m still wondering what’s going on with him [the patient], whether he’s really OK…”*

*“I was given advice that it’s not ethically right to help the patient [financially] but I still needed me as a person I felt I could help…”*

## Learning

Data allocated to the second theme was divided into two sub-themes: (2.1) the impact of supervision on the participants’ learning process, and (2.2) the teaching methods used in supervision.

### Impact of supervision on the participants’ learning process

There was a general consensus that supervision was very important to the student’s learning. It helped them to become more aware of their learning needs, how to meet those learning needs and therefore how to grow as a clinical psychologist.

*“To me, I think the supervision has been, it has made me know myself as a person, this self awareness of my weakness and strengths and also, because of the supervision, it’s made me read a little more and try to be conceptualise all the different theories and see what I’ve realized at a particular time”.*

*“[Supervision] has helped me to know which really approach I can use and even to discover myself which area can really concentrate on or I can specialize so right now I know I consider it a direction I can take.”*

### The methods used to learn in supervision

The participants stated that allowing them to observe therapy, take on a co-therapist role and be observed while conducting therapy were seen as useful ways to learn therapeutic skills. The supervisor telling the students what to do, discussing cases and asking questions also helped the students’ learning.

Two students thought that parallel processes within the supervisory relationship acted as a good model for developing therapeutic relationships with clients. However three of the students stated that this relationship had been unhelpful. They said that their supervisors did not have time for them, which had a negative impact on their ability to learn.

*“We meet and share about a case and then we share notes and say ‘I think this would be beneficial, I think we need to read this’ and then we read about it and then I go apply it next we discuss how it went and I think that’s been really cool”*

*“I go through therapy indirectly so I know how a client would feel for example if he’s not attended to, so that relationship has made me empathetic enough with a client because I can infer from the supervisors that he’s so validating about me so I should be the same with my client.”*

## What supervision had taught the clinical psychology students

The students said that, from supervision, they had learnt about (3.1) their role as a clinical psychology student, (3.2) how to act within the Ugandan mental health system and (3.3) skills in conducting therapy.

### Role as a clinical psychology student

The two main roles discussed were the students’ roles within supervision and on the clinical psychology course. Within supervision, they said it was helpful to know what the supervisor expected of them when they were seeing clients and what was expected of them in supervision.

“*My supervisor informed us that supervision is not all about the patient it is also about her being involved in helping us if we are interested so that made us very comfortable in her hand.”*

With regards to their role on the clinical psychology course, the students said the supervisors helped them to know what the University required of them, such as logging hours of client contact and that it is not necessary to record every session with a client.

“*I learnt that every moment in time I go and meet a patient it counts unto my practicum. Every time you come to Butabika make sure you document what you’ve been able to do on a particular day.”*

### How to act within the Ugandan mental health system

The majority of dialogue in this theme related to learning what was expected of them as a clinical psychologist in the Ugandan mental health system. This included learning which clients were appropriate for psychological therapy, client-therapist boundaries, the importance of not missing appointments with clients, how to practically structure therapy, how to contact clients, and ‘tips of the trade’ such as how to structure day-to-day life as a clinical psychologist and how to approach client files.

“*Ok from my supervisor first and foremost, I learnt client*-*therapist boundaries. Sometimes I would um get taken up in a session and sometimes I would cross my boundaries but I learnt client*-*therapist boundaries.”*

“*From my supervisor I learnt one thing that it’s always better not to look at the file, especially if the client has been referred to you because when you look a the file you’re going to be looking for that diagnosis in the file even when the client has something else, you’re going to be actively looking for symptoms. If the client has PTSD you’re going to you know probe if they have dreams and all and waste time when the client has something it’s not always a client has diagnosis. Some people are misunderstood…I learnt that I should always go into therapy open minded and not even look at the file.”*

### Learning skills in conducting therapy

All the participants agreed that supervision was the main place where practical skills of conducting therapy could be learnt, with the training course emphasising the theoretical elements. The students gave a long list of techniques that supervisors had helped them to learn, including CBT assessment, psycho-education, using outcome measures, giving homework, building therapeutic relationships with clients, making treatment plans, working with specific mental health diagnoses, using translators and working with groups.

*“I didn’t know how to treat, even how to make a treatment plan. But when we met, in fact he first told me about the expectation he thought he had. Thereafter we went through how to make a diagnosis together, and then how to make treatment plan and exactly how to treat”*

*“The supervisor told me no this is what you should address, for example I used to interrupt a lot and when my supervisor told me you should not interrupt, and also because of the fear I think I’ve got this question, I want it to come out and the patient is still talking and I used to be like a robot but out of that the fear reduced and the robot like life also reduced.”*

## Future

The participants discussed ideas for the future of supervision, with this theme being divided into (4.1) general feedback on supervision, (4.2) systems around supervision and (4.3) supervision and the clinical psychology course.

### General feedback on supervision

The participants all said they felt supervision had enhanced their experience of training to be a clinical psychologist, and that it should not only definitely continue for future years, but should start earlier.

*“I think it’s better if it can start earlier, like as soon as you get in contact with clients, like for example the second semester of first year. If we had a supervisor already we would have learnt much more than now”.*

*“Supervision is very very necessary…”*

### Systems around supervision

The students said they had received supervision once a week (n = 3), every fortnight (n = 1), at no set times (n = 2) or once in total (n = 5). They recognised that the heavy workload placed on Ugandan mental health systems had impacted on the availability of the supervisors, and hence their ability to conduct supervision. Some students said that this should be taken into account when selecting supervisors, and only those who can offer weekly supervision should be chosen. They also felt that the supervisors should be briefed before taking on students so that they are aware of what is expected of them, including ensuring that all students gained observational experiences.

The students stated that the clients admitted to the psychiatric hospital, Butabika, presented with acute mental health difficulties. The consensus was that it was difficult to apply the University- taught theories of CBT to these clients. To overcome this barrier, the students suggested for future students to be allowed to have clinical placements in the community or for clinical psychologists to select suitable clients from Butabaika. There was also a request for multi-disciplinary working in the field of clinical psychology for future placements.

*“Sometimes our supervisors have very difficult work schedules and maybe even the supervision is taking a toll on them so they don’t have so much time…definitely creating schedules for other students in the future, it’s good to take into consideration people who are actually available and have enough time because it’s more like a voluntary thing and no*-*one is really getting paid for it but we are benefiting a lot, so we would like someone who has time for us.”*

*“Because you can go to Butabika and then they give you a psychotic full blow patient who cannot be applicable to CBT so I think that other than waiting for us to find a client…[clinical psychologist should] identify the patients and influence to know which clients apply to CBT”*

### Supervision and the clinical psychology course

This sub-theme concerned how the teaching on the Masters programme could better match clinical practice. There was a request for more practical training in a wider variety of approaches. The students stated that they had been taught CBT approaches and had recently received a 2 week external programme in NET. They favoured the practical teaching methods and simplicity used in the NET training, and requested more of the practical-based training. A student asked for psychological therapy to be offered as part of the supervision package in the future.

*“We’ve been through CBT and um it’s a very good well structured therapy that helps a lot in most of the disorders there but, you know when I want for the Narrative [Exposure] Therapy I realized there are also other therapies out there which are more helpful so we could implement even them”*

*“Surely this workshop we had, I wish we were introduced to things that way. Because the whole thing was brought from heaven to earth so much that it was really, really there very practical…how possible will it be to see hands*-*on much like we did…if we are to easily grasp ideas and for the better of the future I think if we handled them just like the way it was done in the NET it took us two weeks on week theory and one week practical, but I mean we learnt a lot of things there than right now I can practice…if we took that approach, we will achieve a little more than the scary approach of reading a big book and you conceptualise so many ideas which look like they are in the sky there but they could be brought on earth and we understand them.”*

## Miscellaneous data

Data not coded for in the previous five themes included students agreeing with each other and them expressing gratitude to the facilitators for running the group.

*“I would like to say thank you for this chance because if gives you feedback and also makes us feel comfortable that you’re concerned with what we are going through.”*

## Discussion

Following a qualitative focus group design, thematic analysis was used to present the Ugandan clinical psychology students’ initial experiences of clinical supervision. The students reflected on the aspects of supervision that had helped them and those that had not, and they suggested changes for future clinical psychology supervision. Aspects voiced as helpful were receiving emotional support, building their own confidence and self awareness, learning how to do therapy practically and understanding expectations of supervision/the course. Learning through discussion, talking about the supervisory relationship, observation of the supervisor and joint working with the supervisor were deemed useful. The not so useful aspects of supervision included the supervisor not addressing student’s emotional issues as much as the student desired and the supervisor not being able to give the student time or observational experiences.

These findings resonate with previous research [36, 42] which suggested that joint problem solving, reassurance and direct guidance on clinical work was useful. The supervisor being unreliable was thought to be unhelpful, which is consistent with previous research (e.g. [34, 35]). The students stated that supervision is helpful when restorative (e.g. emotional support), formative (e.g. expectations of the Masters course) and normative (e.g. therapeutic skills) needs are met, consistent with the functions of supervision proposed (e.g. [25]). Previous Western research suggests that supervisors “telling” the supervisees what to do is seen as unhelpful (e.g. [35, 36]), something which was contradicted in this study, with students finding this helpful. Perhaps the reason for this being that the students were all in the beginning of their development as clinical psychologists and, in line with the developmental model designed by Stoltenberg et al. as cited in [27]) showed a tendency to be more dependent on their supervisor. Hirons and Velleman’s [36] sample stated that talking to the trainee as if they are a client was found unhelpful, whereas psychological therapy within the supervisory relationship was requested in this study. Perhaps this is because very little is known about psychological therapy within the Ugandan culture, and the students wanted to experience therapy themselves.

The clinical psychology students stated that supervision helped them to manage their negative emotions which resulted as a feeling of being overwhelmed by training as clinical psychologist, and that it helped for them to feel more confident in knowing what they were doing (through learning from supervision and the supervisors helping them to manage their anxieties). These two aspects of the results can be compared to the two domains of burn-out emotional exhaustion and reduced personal accomplishment/efficacy [6, 7]. Hence it is possible that supervision helped the clinical psychology students to manage these domains of burn out and hence decreased the likelihood of them experiencing burn out, however more research is needed to ascertain this. Supervision has previously been shown to decrease symptoms of burn out in low income countries [18].

Hence, the finding that the clinical psychology students stated that clinical supervision was useful was in line with the hypotheses.

It was also hypothesised that the useful and not so useful aspects of supervision would be different to those described by Western clinical psychology students due to the differences in culture. However, the results do not support this second hypothesis, with the vast majority of helpful and not so helpful aspects from this study being mirrored in previous Western research. Hence this study suggests that Western “models” can be applied to these initial findings in supervision, however more research is required to determine this.

Limitations in this study include that the clinical psychology students had only received a small number of supervision sessions, and so may not yet be fully aware of the expectations of supervision and be able to critically analyse the useful and not useful aspects. The authors of this paper were all involved in the training and implementation of the supervision at Makerere University, meaning that the students may have felt an implicit need to “please” the facilitators. Furthermore, this may have caused bias in the data analyses stages.

The scope for further research into supervision for clinical psychology students and other mental health professionals in low income countries is vast, with it being suggested that further qualitative research is conducted looking at developmental models, functions of supervision, impact of supervision on burn out and into the useful and not so useful aspects of supervision at different stages of training.

Results from this study suggest that supervision for clinical psychology students in Uganda should continue. Supervisors should focus on being reliable through offering regular time-slots for supervision and they should teach the students the practical elements of psychological therapy through role play, co-therapy and observational experiences. It is vital to ensure that all of the restorative, normative and formative elements of supervision are fulfilled.

The mental wellbeing of practitioners needs to be prioritised in the mental health systems in order to decrease the high levels of burnout and to enable clinical psychology in Uganda to grow as a profession in its own right. This initial research suggests that supervision may be a way to support the emotional needs of clinical psychology students in Uganda. Regular, structured clinical supervisory structures should be set up and evaluated, with the view of this becoming a requirement for accreditation within the Ugandan Clinical Psychology Association. Only once the emotional wellbeing of Ugandan clinical psychologists and other mental health professionals have been addressed can these professionals begin to work securely and safely in this high stress environment. Further research into regular supervision of clinical psychologists, both during training and once qualified, is necessary to ensure that clinical psychology develops in a culturally appropriate manner in Uganda.

## **Additional files**

Additional file 1:
**Table** **S1.** Data excerpt and coding. Additional file 2:
**Table** **S2.** Themes and sub-themes.
